# Antimicrobial Resistance Dynamics in Chilean *Shigella sonnei* Strains Within Two Decades: Role of *Shigella* Resistance Locus Pathogenicity Island and Class 1 and Class 2 Integrons

**DOI:** 10.3389/fmicb.2021.794470

**Published:** 2022-02-04

**Authors:** Cecilia S. Toro, Juan Carlos Salazar, David A. Montero, Juan Antonio Ugalde, Janepsy Díaz, Leandro A. Cádiz, Tania Henríquez, Camila García, Patricia Díaz, Rossanna Camponovo, Germán Hermosilla, María Teresa Ulloa

**Affiliations:** ^1^Programa de Microbiología y Micología, Facultad de Medicina, Instituto de Ciencias Biomédicas, Universidad de Chile, Santiago, Chile; ^2^Programa Disciplinario de Inmunología, Facultad de Medicina, Instituto de Ciencias Biomédicas, Universidad de Chile, Santiago, Chile; ^3^C+, Research Center in Technologies for Society, School of Engineering, Universidad del Desarrollo, Santiago, Chile; ^4^Millennium Initiative for Collaborative Research on Bacterial Resistance, Santiago, Chile; ^5^Departamento de Estudios Científicos, Instituto de Salud Pública de Chile, Santiago, Chile; ^6^Escuela de Tecnología Médica, Facultad de Medicina, Universidad de Chile, Santiago, Chile; ^7^Integramédica-BUPA, Santiago, Chile

**Keywords:** *Shigella sonnei*, antibiotic resistance, mobile genetic elements (MGE), integrons, SRL pathogenicity island, multidrug-resistant (MDR) bacteria, *dfrA14*, class 2 integron

## Abstract

Shigellosis is an enteric infectious disease in which antibiotic treatment is effective, shortening the duration of symptoms and reducing the excretion of the pathogen into the environment. *Shigella* spp., the etiologic agent, are considered emerging pathogens with a high public health impact due to the increase and global spread of multidrug-resistant (MDR) strains. Since *Shigella* resistance phenotype varies worldwide, we present an overview of the resistance phenotypes and associated genetic determinants present in 349 Chilean *S. sonnei* strains isolated during the periods 1995–1997, 2002–2004, 2008–2009, and 2010–2013. We detected a great variability in antibiotic susceptibility patterns, finding 300 (86%) MDR strains. Mobile genetic elements (MGE), such as plasmids, integrons, and genomic islands, have been associated with the MDR phenotypes. The *Shigella* resistance locus pathogenicity island (SRL PAI), which encodes for ampicillin, streptomycin, chloramphenicol, and tetracycline resistance genes, was detected by PCR in 100% of the strains isolated in 2008–2009 but was less frequent in isolates from other periods. The presence or absence of SRL PAI was also differentiated by pulsed-field gel electrophoresis. An atypical class 1 integron which harbors the *bla*_OXA–1_*-aadA1-IS1* organization was detected as part of SRL PAI. The *dfrA14* gene conferring trimethoprim resistance was present in 98.8% of the 2008–2009 isolates, distinguishing them from the SRL-positive strains isolated before that. Thus, it seems an SRL-*dfrA14 S. sonnei* clone spread during the 2008–2009 period and declined thereafter. Besides these, SRL-negative strains harboring class 2 integrons with or without resistance to nalidixic acid were detected from 2011 onward, suggesting the circulation of another clone. Whole-genome sequencing of selected strains confirmed the results obtained by PCR and phenotypic analysis. It is highlighted that 70.8% of the MDR strains harbored one or more of the MGE evaluated, while 15.2% lacked both SRL PAI and integrons. These results underscore the temporal dynamics of antimicrobial resistance in *S. sonnei* strains circulating in Chile, mainly determined by the spread of MGE conferring MDR phenotypes. Since shigellosis is endemic in Chile, constant surveillance of antimicrobial resistance phenotypes and their genetic basis is a priority to contribute to public health policies.

## Introduction

Shigellosis is one of the most common diarrheal diseases in children under 5 years old, especially in developing countries, although it occurs in people of all ages worldwide (Kotloff et al., 2018). *Shigella* spp., the etiologic agent, are considered emerging pathogens with a high public health impact due to the increase and global spread of multidrug-resistant (MDR) strains (Chu et al., 1998; Ashkenazi et al., 2003; Vrints et al., 2009; Shiferaw et al., 2012; Gu et al., 2017; Puzari et al., 2018). This situation is particularly worrying because shigellosis is one of the few enteric infections for which antibiotics are recommended as therapeutic management (Williams and Berkley, 2016, 2018). Antibiotics shorten the severity and duration of symptoms, reducing the excretion time of the pathogen and potential complications of the infection (Chu et al., 1998).

*Shigella* resistance phenotype varies worldwide. The emergence of *Shigella* spp. resistant to the most commonly used antibiotics for treating this disease is reported in Latin America, North America, Europe, and South Asia (Chu et al., 1998; Oh et al., 2003; Gu et al., 2012; Shiferaw et al., 2012; Marcoleta et al., 2013; Puzari et al., 2018; Chung The et al., 2019, 2021; Sati et al., 2019; Bardsley et al., 2020).

The genetic determinants of MDR phenotypes in *Shigella* spp. have been associated with the presence of different plasmids, integrons, and genomic islands (Muñoz et al., 2003; Turner et al., 2003; Toro et al., 2005; Barrantes and Achí, 2016; Miranda et al., 2016; Kang et al., 2019; Ranjbar and Farahani, 2019). The *Shigella* resistance locus pathogenicity island (SRL PAI) is a 66-kb genomic region inserted into the *serX* gene (which codifies for tRNA^Ser^), conferring resistance to ampicillin, streptomycin, chloramphenicol, and tetracycline. A ferric dicitrate transport system and genes involved in D-aspartate metabolism are also encoded in SRL PAI, described firstly in *Shigella flexneri* 2a YSH6000 (SRL_YSH6000_; Luck et al., 2001; Turner et al., 2003; Henríquez et al., 2020). Additionally, integrons have played an essential role in the spread of antimicrobial resistance genes since their discovery in 1989. Integrons are composed of three elements: the *intI* gene that encodes a tyrosine recombinase (integrase), the adjacent recombination site (*attI*) recognized by the integrase, and the promoter (P_c_) necessary for the efficient transcription and expression of gene cassettes present in the integron. They have the ability to capture and express foreign genes, such as resistance gene cassettes, and facilitate their horizontal transfer to a wide range of pathogens (Stokes and Hall, 1989; Recchia and Hall, 1995). Class 1 and class 2 integrons have widely disseminated among species of the family *Enterobacteriaceae* and other Gram-negative bacteria. In *Shigella* spp., antimicrobial resistance is often due to both classes of integrons (Pan et al., 2006; Chang et al., 2011; Kaushik et al., 2018; Shariati et al., 2018; Kang et al., 2019).

The heterogeneous geographical distribution of antimicrobial-resistant strains and mobile genetic elements (MGE) underscores the importance of keeping the local antimicrobial resistance surveillance. Hence, the objective of this study was to characterize the phenotypic and genotypic diversity of Chilean *S. sonnei* that have circulated for almost two decades from 1995 to 2013, focusing on the MDR strains.

## Materials and Methods

### Bacterial Strains and Culture Conditions

**FIGURE 1:**
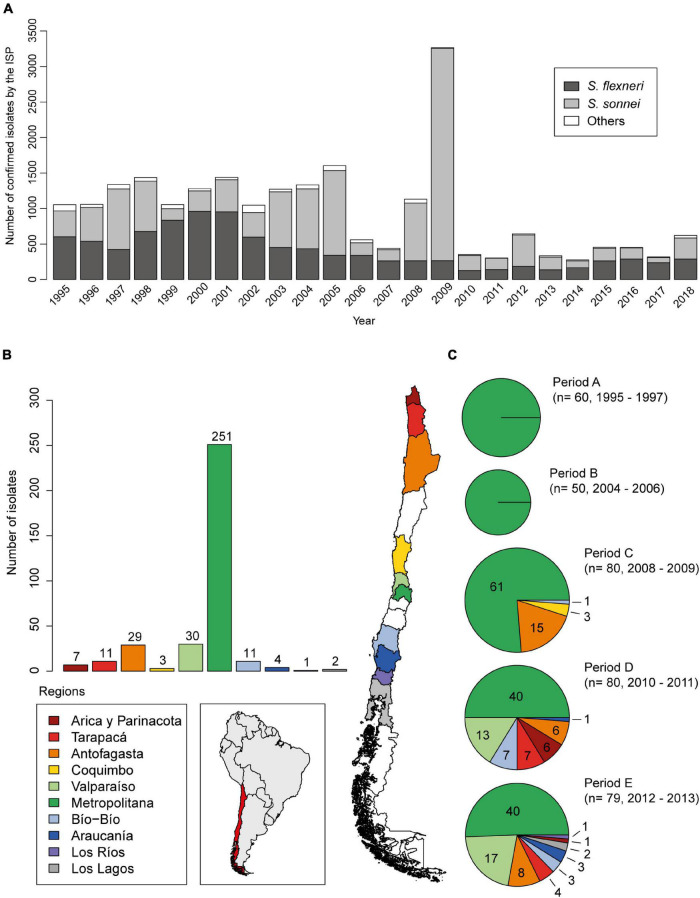
Epidemiological distribution of *Shigella sonnei* in Chile. **(A)** Nationwide detection of *Shigella* spp. strains confirmed by Instituto de Salud Pública de Chile, ISP from 1995 to 2018. **(B)** Geographical distribution of 349 *Shigella sonnei* strains analyzed in this work. **(C)** Characterization of groups defined by time of isolation and geographical distribution. *S. sonnei* strains from periods A and B were isolated from patients living in Region Metropolitana. Period C is composed by strains mostly isolated from the Region Metropolitana (61/80) and from Antofagasta (15/80), a region 1,000 km north from Santiago (Región Metropolitana, Chile). Periods D and E include representative strains from Región Metropolitana (40 in each period) and from the northern and southern regions of the country, which were kindly provided by the ISP **(B,C)**.

A total of 349 *Shigella sonnei* strains were obtained from a bacterial collection belonging to the Programa de Microbiología y Micología, ICBM, Universidad de Chile, isolated from stool samples of patients suffering from acute diarrhea (*n* = 190; 1995–2004) and from the Instituto de Salud Pública de Chile [ISP], 2019 (Public Health Institute of Chile) (*n* = 159; 2010–2013). These strains were grouped into five time periods according to the isolation date: period A, between 1995 and 1997 (*n* = 60); period B, between 2002 and 2004 (*n* = 50); period C, between 2008 and 2009 (*n* = 80); period D, between 2010 and 2011 (*n* = 80); and period E, between 2012 and 2013 (*n* = 79) (Figures 1B,C). *S. sonnei* strains from periods A and B were isolated from patients living in Región Metropolitana. Period C is composed of strains mostly isolated from Región Metropolitana (61/80) and Antofagasta (15/80), a region located 1,000 km north of Región Metropolitana. Periods D and E include representative strains from Región Metropolitana (40 in each period) and northern and southern areas of the country (Figures 1B,C). The isolates were stored at −80°C in trypticase soy broth with 30% glycerol. The *Shigella* strains were identified by conventional and automated biochemical methods (VITEK-2, bioMérieux) and serotyped by agglutination with type-specific antisera (phase I and II, Denka Seiken, Tokyo, Japan). All strains were routinely cultured at 37°C on LB broth or agar.

### Antimicrobial Susceptibility

The antibiotic susceptibility of *S. sonnei* strains was determined by disk diffusion assay tests, performed according to the Clinical and Laboratory Standards Institute guidelines (CLSI, 2018). The antibiotics included the following: ampicillin (AMP), 10 μg; cefotaxime (CTX), 30 μg; chloramphenicol (CHL), 30 μg; tetracycline (TET), 30 μg; streptomycin (STR), 10 μg; sulfamethoxazole/trimethoprim (SXT), 23.75/1.25 μg; trimethoprim (TMP), 30 μg; nalidixic acid (NAL), 30 μg; and ciprofloxacin (CIP), 5 μg. *Escherichia coli* ATCC 25922 was included as control. For purposes of the current analysis, both intermediate and resistant isolates were considered resistant.

### Detection of Integrons and *Shigella* Resistance Locus Pathogenicity Island

The presence of the MGE associated with antibiotic resistance was detected by conventional PCR. DNA templates were obtained from bacterial lysates of individual colonies or genomic DNA preparation (E.Z.N.A. Bacterial DNA kit, Omega, Bio-Tek, Atlanta, United States). Specific primers for class 1 and class 2 integrases were designed (Supplementary Table 1). Two microliters of the sample were added to 20 μl of the reaction mix containing 3 mM MgCl_2_, 400 μM (each) dNTPs (Invitrogen, United States), and primers (25 nM) together with 1 U of *Paq* polymerase (Agilent Technologies, United States). PCR reactions were performed as follows: an initial 2-min denaturation cycle at 95°C, followed by 30 cycles at 95°C for 1 min, 56°C for 30 s, and 72°C for 30 s, with a final extension at 72°C for 10 min. The PCR products were analyzed by electrophoresis in 1% agarose gels and stained with ethidium bromide.

Primers for the detection of SRL PAI were designed using the sequences of the flanking regions of the *serX* gene and the SRL island of *S. flexneri* YSH6000 (GenBank accession number: AF326777.3; Luck et al., 2001). The presence of SRL PAI was defined by three PCR reactions; the presence of SRL PAI (SRL-positive) was defined by the following pattern: SRL I (−), SRL II (+), and SRL III (+). When the PCR pattern was SRL I (+), SRL II (−), and SRL III (−), strains were classified as SRL-negative. Any other amplification pattern was considered also as SRL-negative. The PCR reactions were performed with an initial 2-min denaturation cycle at 95°C, followed by 30 cycles at 95°C for 1 min, 50°C for 30 s, and 72°C for 1 min, with a final extension at 72°C for 10 min. The PCR products were analyzed by electrophoresis in 1.5% agarose gels and stained with ethidium bromide.

### Detection of Antimicrobial Resistance Genetic Markers

DNA templates were obtained as mentioned before. Two microliters of the sample was added to 20 μl of the reaction mix containing 3 mM MgCl_2_, 400 μM (each) dNTPs (Invitrogen, United States), and primers together with 1 U of *Paq* polymerase (Agilent Technologies, United States). Specific primers for trimethoprim resistance genes (*dfrA1*, *dfrA8*, and *dfrA14*), *bla*_OXA–1_ for ampicillin, *cat* for chloramphenicol, and *qacEΔ1* for quaternary ammonium salts resistance were previously described (Supplementary Table 1; Di Conza et al., 2002; Toro et al., 2005; Miranda et al., 2016). The PCR reactions were performed as follows: an initial 2-min denaturation cycle at 95°C, followed by 30 cycles at 95°C for 1 min, 56°C for 30 s, and 72°C for 1 min, with a final extension at 72°C for 10 min. The PCR products were analyzed by agarose gel electrophoresis and stained with ethidium bromide.

### Pulsed-Field Gel Electrophoresis

According to PulseNet protocol (CDC)^1^, bacterial suspensions of 276 *S. sonnei* strains were embedded in agarose plugs, lysed, and then digested with endonuclease *Xba*I (Thermo Fischer Scientific, United States) at 37°C for 2 h. The macrorestriction of genomic DNA fragments was separated by pulsed-field gel electrophoresis on a CHEF-DRIII Chiller system (Bio-Rad Laboratories, Richmond, CA, United States) in 1% agarose gel using 0.5X TBE buffer at 6 V/cm and 14°C, with ramped pulse times of 2.2–54.2 s for 21 h. *Salmonella enterica* serovar Braenderup strain H9812 was included as molecular size standard three times on each gel to normalize the images and to compare the fingerprints among several gels. The DNA band profiles were analyzed with GelCompar software (version 3.0; Applied Maths, Sint-Martens-Latem, Belgium). A similarity dendrogram was constructed using the unweighted-pair group method with arithmetic mean (UPGMA) with the Dice similarity coefficient and a band tolerance of 1.5%. Pulsegroups were defined by sharing 73% similarity and more than 93.5% for pulsetypes.

### Genome Sequencing and Data Processing

Illumina sequencing was performed on 29 selected Chilean *S. sonnei* strains by Microbes NG (Birmingham, United Kingdom) using the Nextera XT library protocol. Sequencing was done on the Illumina MiSeq platform (Illumina, San Diego, CA, United States). Reads were processed with Trimmomatic 0.30 to remove adapters and for quality trimming. For *de novo* genome assembly, reads were processed using Shovill^2^ with Spades 3.14.0 (Nurk et al., 2013)^3^, with a Kmer range from 31 to 127 (31, 55, 79, 103, and 127). For SNP identification, the trimmed reads were mapped to the reference genome of *S. sonnei* SS046 using Snippy with default parameters^4^. The core SNPs were identified from the alignment, and a final phylogenetic tree was generated using FastTree (Price et al., 2010). Resfinder was used to predict the presence of known acquired resistance genes and chromosomal mutations associated with resistance to quinolones (Camacho et al., 2009; Bortolaia et al., 2020; Zankari et al., 2020)^5^. MLST typing using sequences was done by https://cge.cbs.dtu.dk/services/MLST/ (Larsen et al., 2012). All the assembled genomes from this study are available on NCBI under the BioProject accession PRJNA602693.

### Comparative Genomic Analysis

Draft genome sequences were managed using the Geneious software (v11.0.5; Biomatters, Ltd., New Zealand). The assembled contig sequence was mapped against the reference sequence of the SRL PAI carried by *S. flexneri* 2a str YSH6000 using BlastN in Geneious. Comparisons of the genetic structure of SRL PAIs were performed using EasyFig v2.1 (Sullivan et al., 2011).

### Statistical Analysis

Evaluation of pairwise association between the timeframe of isolation of the strains and the specific genes that they carry was performed using contingency tables by odds ratios. The statistical significance of these associations was determined by Pearson’s chi-square test or Fisher’s exact test (when frequencies were less than 5) (R Core Team, 2014; Csardi, 2015; Wei et al., 2017). When any of the cell values of the contingency table was zero, Haldane correction (Haldane, 1940) was used by adding 0.5 to all cells.

## Results

### Antibiotic Resistance Phenotypes of Chilean *S. sonnei* Strains

To characterize the molecular and phenotypic resistance profile of Chilean *S. sonnei* strains, we analyzed 349 available strains isolated between the years 1995 and 2013. To better understand the temporal dynamics of antibiotic resistance of these strains, they were grouped according to the date of isolation in periods A–E. This collection included a total of 80 strains isolated in 2008–2009, which corresponds to period C, when an increase of *S. sonnei* frequency was registered (Figure 1A). The geographical origin of the strains is shown in Figures 1B,C.

Antimicrobial susceptibility test (AST) for all these 349 strains showed that 100% were sensitive to ciprofloxacin, while 3 (0.9%) and 33 (9.5%) isolates were resistant to cefotaxime and nalidixic acid, respectively. In contrast, these strains displayed high rates of antibiotic resistance to streptomycin (305, 87.4%), trimethoprim (264, 75.6%), ampicillin (257, 73.6%), tetracycline (257, 73.6%), sulfamethoxazole/trimethoprim (256, 73.3%), and chloramphenicol (195, 55.9%). The resistant phenotype to the last five antibiotics in period C is particularly noteworthy (Table 1).

**TABLE 1:** Antimicrobial resistance of Chilean *Shigella sonnei* strains from 1995 to 2013.

Antimicrobial agent	Periods					
	A (1995–1997)	B (2002–2004)	C (2008–2009)	D (2010–2011)	E (2012–2013)	Total (1995–2013)
Streptomycin	60 (100)	50 (100)	80 (100)	51 (63.8)	64 (81)	305 (87.4)
Trimethoprim	28 (46.7)	27 (54)	80 (100)	61 (76.3)	68 (86.1)	264 (75.6)
Ampicillin	52 (86.7)	31 (62)	80 (100)	53 (66.3)	41 (51.9)	257 (73.6)
Tetracycline	27 (45)	17 (34)	80 (100)	64 (80)	69 (87.3)	257 (73.6)
Sulfamethoxazole/trimethoprim	28 (46.7)	25 (50)	80 (100)	57 (71.3)	66 (83.5)	256 (73.4)
Chloramphenicol	25 (41.7)	1 (2)	80 (100)	53 (66.3)	36 (45.6)	195 (55.9)
Nalidixic acid	0 (0)	0 (0)	0 (0)	1 (1.3)	32 (40.5)	33 (9.5)
Cefotaxime	0 (0)	0 (0)	0 (0)	1 (1.3)	1 (1.3)	3 (0.9)
Ciprofloxacin	0 (0)	0 (0)	0 (0)	0 (0)	0 (0)	0 (0)

*The number of strains analyzed by period are described as follows: A = 60, B = 50, C = 80; D = 80, and E = 79. The number of strains and percentage in parenthesis are shown for each cell.*

Isolates resistant to at least one antibiotic in three or more drug classes were considered MDR^6^. Consequently, 300 out of 349 strains (86%) presented an MDR phenotype. Furthermore, the most frequent MDR phenotype was AMP-CHL-TET-STR-SXT-TMP, displayed by 123 strains (35.2%), followed by AMP-CHL-TET-STR (36, 10.3%), AMP-STR-SXT-TMP (32, 9.2%), TET-STR-SXT-TMP (25, 7.2%), and AMP-CHL-TET-SXT-TMP (20, 6.7%) (Supplementary Table 2).

**FIGURE 2:**
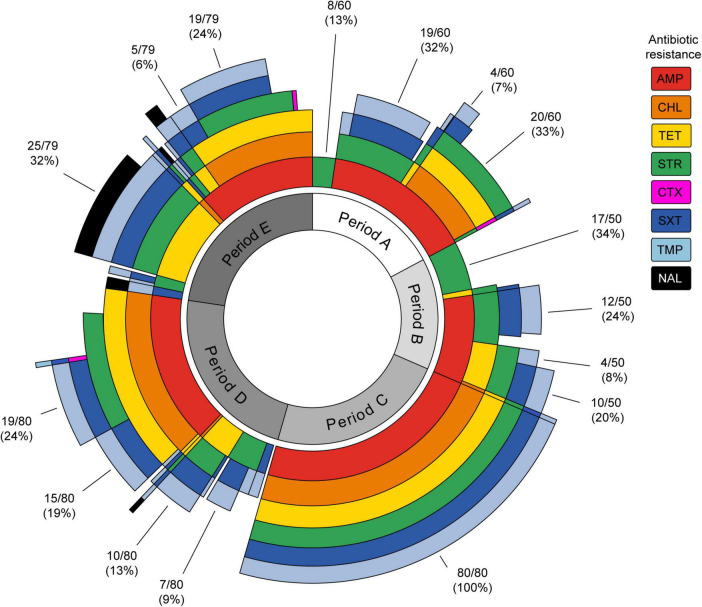
Sunburst plot showing the antibiotic resistance and multi-drug resistance phenotypes of *Shigella sonnei* strains isolated in different time periods. Each antibiotic is represented by a concentric circle of a different color. The sum of the concentric circle represents the resistance phenotype pattern for each strain. The percentages of most frequent profiles for each period are shown. AMP, ampicillin; CHL, chloramphenicol; TET, tetracycline; STR, streptomycin; CTX, cefotaxime; SXT, sulfamethoxazole/trimethoprim; TMP, trimethoprim; NAL, nalidixic acid. The figure was prepared using the sunburstR (Bostock et al., 2019) package in R (R Core Team, 2014).

Figure 2 shows the temporal distribution of the resistance phenotypes by antibiotic (concentric circles) and MDR phenotypes simultaneously. It should be noted that all the strains of period C displayed resistance to six antibiotics (AMP-CHL-TET-STR-SXT-TMP). As mentioned above, this was the most frequent MDR profile in the present study. Interestingly, this MDR phenotype was observed with a lower frequency not only before period C (5/349, 1.4%) but also after this (38/349, 10.9%). On the contrary, in the other periods, heterogeneous MDR phenotypes were observed. Analyzing each period, the most frequent MDR phenotypes in period A were AMP-CHL-TET-STR (20/60, 33%) and AMP-STR-SXT-TMP (19/60, 32%). In period B, AMP-STR-SXT-TMP (12/50, 24%) and AMP-TET-STR-SXT-TMP (10/50, 20%) were detected. Meanwhile, these were AMP-CHL-TET-STR-SXT-TMP (19/80, 24%) and AMP-CHL-TET-SXT-TMP (15/80, 19%) in period D and TET-STR-SXT-TMP-NAL (25/79, 32%) and AMP-CHL-TET-STR-SXT-TMP (19/79, 24%) in period E. Notably, 32 out of 33 NAL^R^ strains were isolated in period E, and 25 of them displayed the TET-STR-SXT-TMP-NAL MDR phenotype, suggesting the emergence of a new clone of *S. sonnei* at that time.

### Distribution of Genetic Elements Associated to Multidrug-Resistant in Chilean *S. sonnei* Strains

The simultaneously resistant phenotype to ampicillin, streptomycin, chloramphenicol, and tetracycline in some *S. sonnei* strains suggested the presence of the SRL PAI. To determine whether these strains harbor this genetic element, three PCR assays were designed. A combination of SRL I, SRL II, and SRL III PCR amplification patterns allowed us to detect the insertion of SRL PAI within the *serX* gene. The SRL I (−), SRL II (+), and SRL III (+) pattern was expected for SRL-positive strains. Conversely, the SRL I (+), SRL II (−), and SRL III (−) pattern was expected for no insertion at *serX*, indicating the absence of SRL PAI.

**FIGURE 3:**
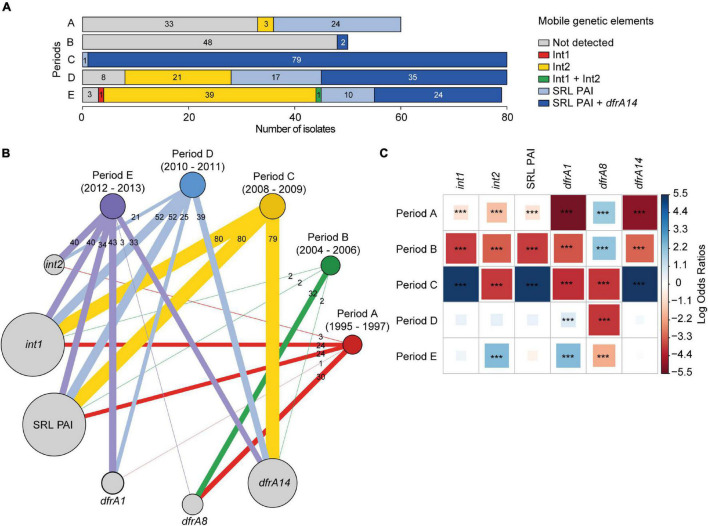
Patterns of association between the *Shigella sonnei* isolation date (periods A–E) and genetic elements related to antimicrobial resistance. **(A)** Distribution of class 1, class 2 integrons, SRL PAI, and *dfrA14* gene. **(B)** Graph of modules showing the interactions between periods of isolation and specific genetic determinants. Modules are weighted by the number of strains. Besides this, links are weighted by both the number of strains linked between modules and the number of strains within modules. The figure was prepared using the igraph (Csardi, 2015) package in R (R Core Team, 2014). **(C)** Pairwise association plot for periods of isolation and specific genetic elements. Red and blue squares represent negative and positive associations, respectively. The color scale represents the magnitude of the association determined by odds ratios. ***p < 0.001 determined by Pearson’s chi-square test or Fisher’s exact test. The figure was prepared using the package corrplot (Wei et al., 2017) in R (R Core Team, 2014).

Of the 349 strains tested, 192 (55%) were classified as SRL-positive and 157 (45%) as SRL-negative. The 192 SRL-positive strains were distributed as follows: 24/60 (40%) from period A, 2/50 (4%) from period B, 80/80 (100%) from period C, 52/80 (65%) from period D, and 34/79 (43%) from period E (Figure 3A). The detailed SRL PAI distribution in the strains is described in Supplementary Table 2. The group of SRL-positive strains includes five strains that should have been classified as SRL-negative due to the PCR pattern. Nonetheless, because their antibiotic profile corresponded to the SRL-positive phenotype, we further evaluated the presence of pathogenicity island integrase and *orf58* genes as well as antibiotic resistance determinant genes in these strains (Supplementary Table 1). The results allowed us to confirm the presence of SRL PAI probably at another insertion site (SRL + out) (data not shown). Any other PCR pattern was considered negative for the presence of SRL, which was obtained for 14 strains (Supplementary Table 2).

In addition, class 1 and class 2 integrons were searched by PCR, using specific primers for integrases (Supplementary Table 1). Class 1 integron was detected in 194/349 (55.6%) strains, including 100% of the SRL-positive strains (Figure 3B). This result is consistent with the presence of an atypical class 1 integron within SRL PAI, which harbors the *bla*_OXA–1_*-aadA1-IS1* gene organization. Indeed, we assessed and detected the adjacent *bla*_OXA–1_ and *aadA1* genes by PCR for at least 70 SRL-positive strains (data not shown).

Of the 157 SRL-negative strains, only one SRL-negative strain from period E harbored a typical class 1 integron (0.3%) (Figure 3A), defined by the *dfrA1-aadA1-qacE*Δ*1-sul1* gene cluster and further confirmed by PCR and whole-genome sequencing (WGS) of the strain.

On the other hand, class 2 integron was detected in 64/349 (18.3%) strains; all of them were SRL-negative isolates. They belong to the following periods: 3 to period A, 21 to period D, and 40 to period E, with one of them harboring class 1 and class 2 integrons simultaneously (Figure 3A). It should be noted that class 2 integron was neither detected among strains from period B nor C (Figure 3B). In addition, 92 isolates (26.4%) were negative for MGE, SRL PAI, and integrons. Of this group, 53 isolates (15.2%) were MDR.

It is interesting to remark that 144 out of 192 (75%) SRL-positive strains displayed an MDR phenotype to AMP-CHL-TET-STR and also to SXT and TMP. Therefore, we searched for the presence of previously detected *dfrA1*, *dfrA8*, and *dfrA14* genes, which confer resistance to TMP (TMP^R^) (Miranda et al., 2016). Overall, *dfrA1, dfrA8*, and *dfrA14* genes were detected in 69 (19.7%), 65 (18.6%), and 153 (43.8%) strains, respectively (Figure 3B). Focusing on the 264 TMP^R^ strains, we detected *dfrA1* in 54 strains (20.5%), *dfrA8* in 51 (19.3%) strains, and *dfrA14* in 142 (53.8%) strains as sole TMP^R^ genetic marker. The combinations *dfrA1-dfrA8*, *dfrA1-dfrA14*, and *dfrA8*-*dfrA14* were scarcely present in 1.1% (3), 3.8% (10), and 0.4% (1), respectively. Three TMP^R^ strains did not carry any of these TMP gene markers, while two TMP-sensitive strains harbored *dfrA1* cassette, and 10 carried *dfrA8* (Supplementary Table 2).

Notably, 140 out of 144 (97.2%) SRL-positive TMP^R^ strains harbored the *dfrA14* gene, distributed as follows: 2 strains in period B, 79 in C, 35 in D, and 24 in E. From the four remaining strains, two of them harbored *dfrA8*, and two lacked the tested TMP^R^ genes. On the other hand, there were 120 SRL-negative TMP^R^ strains that harbored a unique TMP^R^ gene, distributed as follows: 54 with *dfrA1*, 49 with *dfrA8*, and 4 with *dfrA14*. The presence of *dfrA1-dfrA8* or *dfrA1-dfrA14* pairs was detected in 3 and 9 strains, respectively, and one strain was negative for these markers. In addition, the presence of *dfrA1* gene was detected among 62 out of 64 class 2 integron-positive strains.

All these associations are illustrated in Figure 3B, where it can be seen that the distribution of these genetic determinants depends on the isolation periods. In this context, strains from periods A and B were significantly associated with the presence of the *dfrA8* gene; the presence of the SRL PAI, the atypical class 1 integron, and the *dfrA14* gene was associated with strains from period C, while the presence of the class 2 integron and the *dfrA1* gene was associated with strains from period E (Figure 3C).

In summary, we detected 300 (86%) MDR strains out of 349. They were distributed in 247 (70.8%) containing one or more of the MGE evaluated in this work. In contrast, only 53 (15.2%) were negative for SRL PAI and class 1 and 2 integrons.

### Pulsed-Field Gel Electrophoresis Analysis of Chilean *S. sonnei* Strains

**FIGURE 4:**
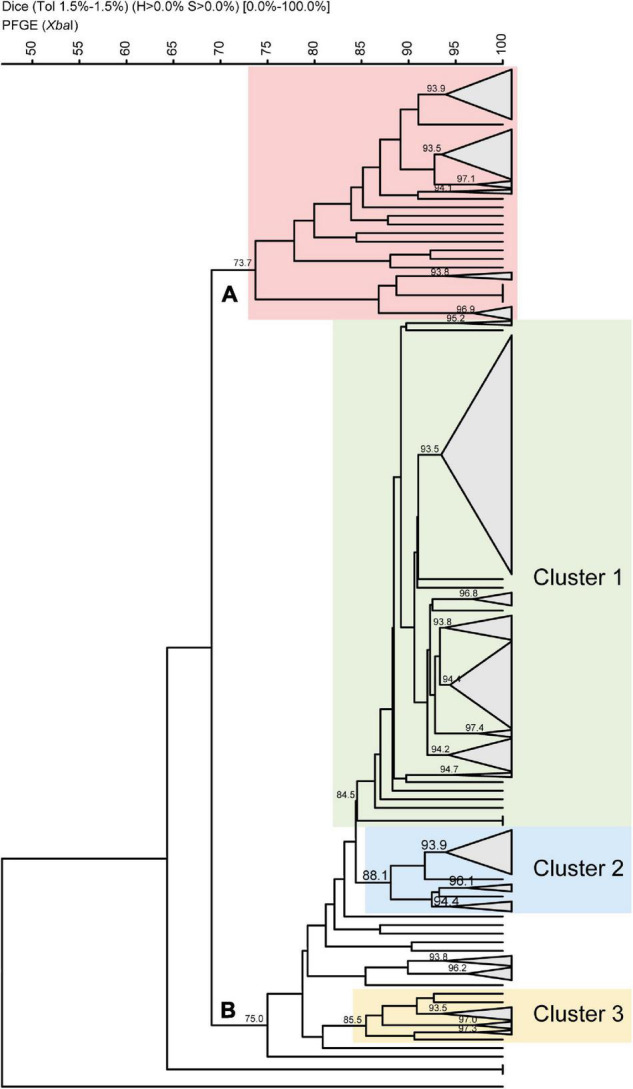
Dendrogram based on Dice coefficients of similarity for 276 Chilean *Shigella sonnei* strains by pulsed-field gel electrophoresis patterns. The dendrogram was constructed by UPGMA (GelCompar II). Shown are two pulsegroups **(A,B)** with a similarity of 69.1%. Twenty-two pulsetypes were defined with a similarity of >93.5%. Within pulsegroup A, all the strains belong to periods A and B, and most of them harbored *dfrA8* as TMP resistance marker. Pulsegroup B comprises 214 strains divided in three clusters. Within cluster 1, there are 144 SRL and SRL/*dfrA14* strains. Cluster 2 contains 25 strains, of which 21 are NAL-resistant strains harboring class 2 integron. Cluster 3 is composed of 13 strains, of which 11 carry class 2 integron, but they are NAL-sensitive strains.

To assess the clonality of the strains in this study, we performed a pulsed-field gel electrophoresis analysis of 276 *S. sonnei* isolates using *Xba*I digestion. The samples covering all the periods were randomly selected, including 56 from period A, 36 from period B, 71 from period C, 48 from period D, and 65 from period E. The obtained tree was split into two major pulsegroups with 69.1% of similarity (Figure 4). Pulsegroup A comprises 62 strains with similarities greater than 73.7% and whose common feature was the absence of SRL PAI. In addition, 55 isolates out of 62 belonged to periods A and B, i.e., strains isolated between 1995 and 2004 (88.7%), and 42 of them (67.7%) carried the genetic marker *dfrA8*.

Pulsegroup B includes 214 strains sharing 75% similarity, and it represents a more heterogeneous group with 3 subgroups or clusters (Figure 4). The largest subgroup, named cluster 1, includes 158 strains sharing 84.5% of similarity. Here 144 out of the 163 tested SRL-positive strains were grouped, with 108 of them carrying SRL/*dfrA14* and 36 just SRL. Cluster 1, the largest pulsetype defined at 93.5% similarity, included 84 strains: 70 isolates carried SRL/*dfrA14*, 10 strains harbored only SRL PAI, and 4 strains were without the island, regardless of the collection period. A subgroup composed of 25 strains (cluster 2) includes 23 isolates harboring the class 2 integron, 21 of which are simultaneously resistant to nalidixic acid. Finally, there is a subgroup composed of 13 strains (cluster 3), from period E, 11 of which have a class 2 integron. Hence, both clusters B2 and B3 include 75.6% of class 2 integron-positive strains and 84% of nalidixic acid-resistant strains of this analysis.

### Whole-Genome Sequence Analysis From Chilean *S. sonnei* Strains

A total of 29 isolates representing different patterns of resistant phenotypes were selected for sequencing. Strains from all periods, positive and negative for SRL PAI and class 1 or 2 integrons, were included. Genetic determinants of resistance were obtained by Resfinder (Bortolaia et al., 2020) and were concordant with phenotypical AST and PCR results (Table 2). Seventeen out of 29 sequenced strains harbored SRL PAI: 11 were SRL/*dfrA14*-positive, and the remaining 6 strains carried only SRL PAI. The presence of the *bla*_OXA–1_, *aadA1*, *cat*, and *tetB* genes in those strains was confirmed. In those SRL/*dfrA14*-positive strains, this gene was harbored in the pABC-3 plasmid. In all cases, disruption of the *strA* gene by the insertion of *dfrA14*, included in the *sul2*/*strA*::*dfrA14*/*strB* genes cluster, was identified. In addition, the *dfrA15* gene was detected as another determinant of TMP^R^ through sequencing of one of the three TMP^R^ strains negative for the other alleles (Table 2).

**TABLE 2:** Phenotypic and genotypic characterization of 29 WGS Chilean *Shigella sonnei* strains.

						Antibiotic susceptibility profiles									PCR detection						WGS		WGS predicted antibiotic resistance genes^a^														
Period	Strain	Refseq	assembly	accession	Origin region	Isolation year	PFGE	AMP	CTX	CHL	TET	STR	SXT	TMP	NAL	CIP	Class 1 *int1*	Class 2 *int2*	SRL PAI	*dfrA1*	*dfrA8*	*dfrA14*	WGS-predicted MLST^b^	Lineage	classification	*blaOXA*	*blaTEM*	*cat*	*cmlA1*	*tetB*	*tetA*	*aadA1*	*strA*	*strB*	*sul1*	*sul2*	*dfrA1*	*dfrA8*	*dfrA14*	*dfrA15*
A	a0044	GCF_019732475.1	Metropolitana	1995	+	R	S	S	S	R	S	R	S	S	–	+	–	–	+	–	ST-152	IIa	–	+	–	–	–	–	–	+	+	–	+	–	+	–	–
A	a0047	GCF_019732435.1	Metropolitana	1995	+	R	S	R	R	R	R	R	S	S	+	–	+	–	–	–	ST-152	IIIa	+	–	+	+	+	–	+	+	+	+	+	–	–	–	+
A	a0148	GCF_019732455.1	Metropolitana	1996	+	R	S	R	R	R	S	S	S	S	+	–	+	–	–	–	ST-152	IIIa	+	–	+	–	+	–	+	+	+	–	+	–	–	–	–
A	a0157	GCF_019732395.1	Metropolitana	1996	+	R	S	S	R	R	R	R	S	S	–	+	–	+	+	–	ST-152	IIIa	–	+	–	–	–	+	–	+	+	–	+	–	+	–	–
A	a0189	GCF_019732375.1	Metropolitana	1997	+	R	S	R	R	R	S	S	S	S	+	–	+	–	–	–	ST-152	IIIa	+	–	+	–	+	–	+	+	+	–	+	–	–	–	–
A	a0195	GCF_019732415.1	Metropolitana	1997	+	R	S	S	S	R	R	R	S	S	–	–	–	–	+	–	ST-152	IIb	+	+	–	–	–	–	–	+	+	–	+	–	–	–	–
A	a0214	GCF_019732315.1	Metropolitana	1997	+	R	S	R	R	R	R	R	S	S	+	–	+	–	+	–	ST-152	IIIa	+	+	+	–	+	–	+	+	+	–	+	–	+	–	–
B	b0061	GCF_019732335.1	Metropolitana	2005	+	R	S	S	R	R	R	R	S	S	+	–	+	–	+	+	ST-152	IIIa	+	+	+	–	+	+	+	+^c^	+	–	+	–	+	+	–
B	b0074	GCF_019732345.1	Metropolitana	2004	+	S	S	S	S	R	S	S	S	S	–	–	–	–	–	–	ST-152	V	–	–	–	–	–	–	–	+	+	–	+	–	–	–	–
B	b0200	GCF_019732295.1	Metropolitana	2006	+	R	S	R	R	I	R	R	S	S	+	–	+	–	–	+	ST-152	IIIa	+	–	+	–	+	–	+	+^c^	+	–	+	–	–	+	–
B	b0566	GCF_019732275.1	Metropolitana	2005	+	R	S	S	R	R	R	R	S	S	–	–	–	–	+	–	ST-152	IIIa	–	+	–	–	–	–	–	+	+	–	+	–	+	–	–
C	c0725	GCF_019732245.1	Antofagasta	2008	+	R	S	R	R	I	R	R	S	S	+	–	+	–	–	+	ST-152	IIIa	+	+	+	–	+	+	+	+^c^	+	–	+	–	+	+	–
C	c0736	GCF_019732235.1	Antofagasta	2008	+	R	S	R	R	I	R	R	S	S	+	–	+	–	–	+	ST-152	IIIa	+	–	+	–	+	–	+	+^c^	+	–	+	–	–	+	–
C	c8225	GCF_019732175.1	Metropolitana	2009	+	R	S	R	R	R	R	R	S	S	+	–	+	–	–	+	ST-152	IIIa	+	–	+	–	+	–	+	+^c^	+	–	+	–	–	+	–
C	c8763	GCF_019732185.1	Metropolitana	2009	+	R	S	R	R	R	R	R	S	S	+	–	+	–	–	+	ST-152	IIIa	–	–	+	–	+	–	+	+^c^	+	–	+	–	–	+	–
C	c8852	GCF_013821725.1	Metropolitana	2009	+	R	S	R	R	R	R	R	S	S	+	–	+	–	–	+	ST-152	IIIa	+	–	+	–	+	–	+	+^c^	+	–	+	–	–	+	–
C	c8870	GCF_019732195.1	Metropolitana	2009	+	R	S	R	R	I	R	R	S	S	+	–	+	–	–	+	ST-152	IIIa	+	–	+	–	+	–	+	+^c^	+	–	+	–	–	+	–
D	d0150	GCF_019732155.1	Bío Bío	2011	+	S	S	S	S	S	R	R	S	S	–	–	–	–	–	+	ST-152	IIIa	–	–	–	–	–	–	–	+^c^	+	–	+	–	–	+	–
D	d0237	GCF_019732135.1	Metropolitana	2010	+	R	S	R	R	R	R	R	S	S	+	–	+	–	–	+	ST-152	IIIa	+	–	+	–	+	–	+	+^c^	+	–	+	–	–	+	–
D	d0930	GCF_019732105.1	Valparaíso	2010	–	R	S	S	R	S	R	R	S	S	–	+	–	+	–	–	ST-152	IIIb	–	–	–	–	–	–	+	+	+	–	+	+	–	–	–
D	d1397	GCF_019732095.1	Arica y Parinacota	2011	+	R	S	R	R	S	R	R	S	S	+	–	+	–	–	+	ST-152	IIIa	+	–	+	–	+	–	+	+^c^	+	–	+	–	–	+	–
D	d1533	GCF_019732055.1	Metropolitana	2011	+	R	S	R	S	S	S	R	S	S	+	–	+	–	–	+	ST-152	IIIa	+	–	+	–	+	–	+	+^c^	+	–	+	–	–	+	–
D	d2078	GCF_019732025.1	Valparaíso	2011	+	S	S	S	S	R	S	R	S	S	–	+	–	+	–	–	ST-152	IIIb	–	–	–	–	–	–	+	–	–	–	–	+	–	–	–
D	d4511	GCF_019732015.1	Metropolitana	2010	–	S	S	S	R	R	R	R	S	S	–	+	–	+	–	–	ST-152	IIIb	–	–	–	–	–	–	+	+	+	–	+	+	–	–	–
E	e0300	GCF_019732075.1	Los Lagos	2012	+	S	S	S	S	S	I	R	R	S	–	+	–	+	–	–	ST-152	Global	–	–	–	–	–	–	–	–	–	–	–	+	–	–	–
E	e1006	GCF_019731995.1	Metropolitana	2012	+	I	S	S	R	R	R	R	R	S	–	+	–	+	–	–	ST-152	Global	–	–	–	–	–	+	–	+	+	–	+	+	–	–	–
E	e1087	GCF_019731955.1	Valparaíso	2013	+	R	S	R	R	S	S	S	S	S	+	–	+	–	–	–	ST-152	IIIa	+	–	+	–	+	–	+	–	–	–	–	–	–	–	–
E	e1409	GCF_013821745.1	Antofagasta	2012	–	R	I	R	R	S	S	S	S	S	+	–	+	–	–	–	ST-152	IIIa	+	–	+	–	+	–	+		–	–	–	–	–	–	–
E	e1660	GCF_019731975.1	Antofagasta	2013	+	R	S	S	R	R	R	R	S	S	+	–	–	+	–	–	ST-1504	Global	–	+	–	–	–	+	+	+	+	+	+	+	–	–	–

*^a^Predicted by ResFinder 4.1 tool (https://cge.cbs.dtu.dk/services/ResFinder/).*

*^b^Predicted by MLST 2.0 tool (https://cge.cbs.dtu.dk/services/MLST/).*

*^c^strA gene is interrupted by the insertion of dfrA14s.*

**FIGURE 5:**
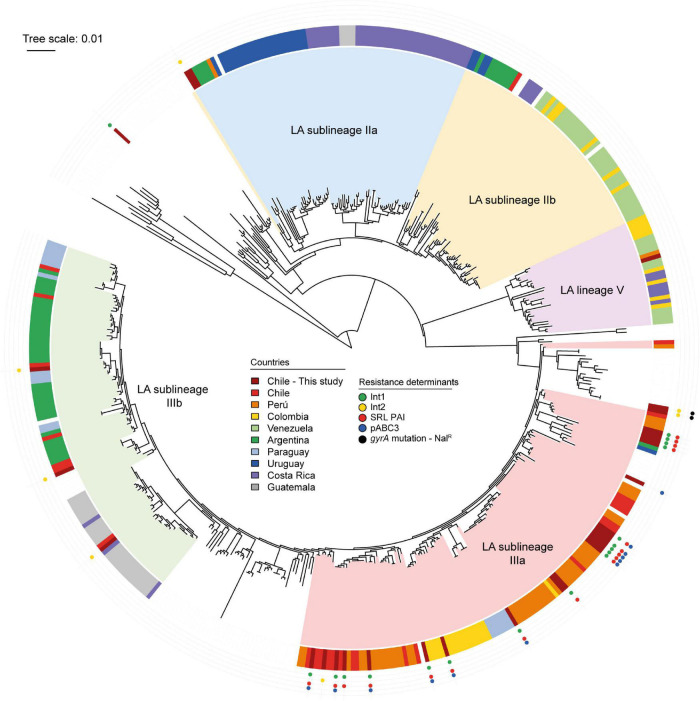
Phylogenetic analysis of *Shigella sonnei* genomes sequenced in this study and publicly available *S. sonnei* genomes. Maximum likelihood phylogenetic tree (midpoint rooted) based on core genome SNPs showing phylogenetic relationships among 470 *S. sonnei* strains. The Latin-American (LA) lineages are based in the study by Baker et al. (2017). The country of origin of LA strains is shown in the colored ring, according to the legend. The global reference strains in the tree do not have country labels. The presence of antimicrobial resistance determinants of Chilean *S. sonnei* strains sequenced in this study is shown in the outer tracks, according to the legend. The tree was edited using iTOL (Letunic and Bork, 2016).

The lineage of the strains was determined according to the location of the genome in the full-core SNP phylogeny, comparing 470 publicly available *S. sonnei* sequences (Figure 5). This analysis allowed categorizing 20 out of 29 Chilean *S. sonnei* strains as sublineage IIIa. In fact, all SRL-positive strains sequenced in this study were sublineage IIIa. The seven sequenced strains harboring the class 2 integron were more heterogeneous, being part of sublineages IIa, IIIa, and IIIb and global. Three class 2 integron-positive strains were classified in sublineage IIIb, sharing this classification with most of the Argentinian strains. Two NAL^R^-sequenced strains, displaying a D87Y mutation in *gyrA* and class 2 integron, belonged to the global lineage (Table 2 and Figure 5).

Using the MLST 2.0 tool, we found that all but one of the sequenced strains had sequence type ST-152 (Table 2). The exception was a strain isolated in period E, whose sequence type was ST-1504. This strain belonged to the global sublineage and was confirmed to be the only one harboring the typical class 1 integron mentioned above.

### Genomic Organization of *Shigella* Resistance Locus Pathogenicity Island in *Shigella*

**FIGURE 6:**
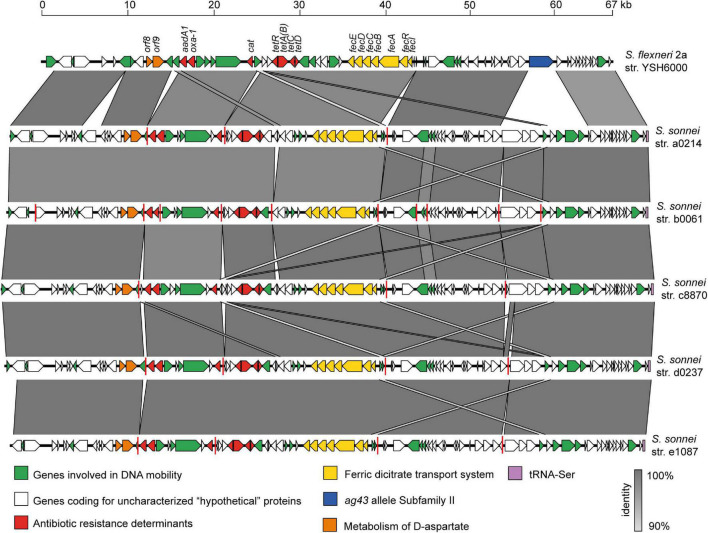
Comparison of the genetic structure of SRL pathogenicity islands carried by the *Shigella flexneri* 2a YSH6000 strain and Chilean *S. sonnei* strains. The predicted genes and the direction of transcription are represented as block arrows. The genes are color-coded according to gene function, as indicated in the legends at the bottom. The names of some genes are indicated. The conserved regions are shaded in gray, and the intensity of the color indicates the nucleotide identity levels, from 88 to 100%. Contig boundaries are shown as red lines. The GenBank accession numbers are indicated in Table 2. The figure was prepared using EasyFig (Sullivan et al., 2011).

Since the SRL PAI was widely distributed among Chilean *S. sonnei* strains isolated over a period of 18 years, we investigated the genetic conservation of this PAI. For this, we performed alignments between the prototypical SRL PAI harbored by the *S. flexneri* 2a str. YSH6000 (SRL_YSH6000_) and five draft genomes of *S. sonnei* representative strains of each period (see section “Materials and Methods”). As a result, we found several contigs containing DNA regions with >90% identity with SRL_YSH6000_. *In silico* analyses allowed us to map these homologous sequences against the genetic structure of SRL_YSH6000_, assembling the complete genetic structure of this PAI in those five analyzed genomes (Figure 6). The SRL PAIs harbored by the *S. sonnei* strains comprise approximately 75 kb. As expected, in all five draft genomes, there is a high degree of conservation compared with the one in the *S. flexneri* YSH6000 strain. Among the genes identified in these PAIs, we found the antibiotic resistance genes (*bla*_OXA–1_, *aadA1*, *cat*, and *tet*), a ferric dicitrate transport system, and the recently described *orfs* 8 and 9 that participate in the metabolism of D-aspartate (Henríquez et al., 2020). However, a significant difference observed in the SRL PAI identified in the *S. sonnei* strains corresponds to a deletion of a gene that encodes an autotransporter protein of the Ag43 allele family. One possible explanation is that this deletion originates from the insertion of a DNA fragment of about 10 kb, containing genes encoding for integrases and hypothetical proteins.

## Discussion

According to data from ISP, the epidemiology of shigellosis in Chile registered around 1,000 *Shigella* isolates per year between 1995 and 2008, from which 47% corresponded to *S. flexneri* and 47% were *S. sonnei*. In contrast, in 2009, there was an increase of up to 3,000 isolates, when a *S. sonnei* peak was observed rising up to about 90%. Furthermore, a sharp decline to less than 500 isolates during the years 2010–2018 was registered. Nevertheless, *S. sonnei* and *S. flexneri* frequency remained near to 50% (ISP, Boletín Vigilancia de Laboratorio, *Shigella* 2014–2018; Figure 1A).

The ISP reports also showed the antibiotic resistance profiles. However, the genetic determinants of resistance in our country have barely been studied. In this context, we described SRL PAI as one of the MGE present in Chilean *S. sonnei* strains. This genetic element was first described in *S. flexneri* 2a YH6000 (Luck et al., 2001). The 66-kb SRL PAI includes an SRL locus encoding resistance determinants to streptomycin (*aadA1*), ampicillin (*bla*_OXA–1_), chloramphenicol (*cat*), and tetracycline (*tetB*). The SRL PAI is likely acquired due to horizontal gene transfer underscoring the role of this mechanism to disseminate *Shigella* MDR phenotype (Turner et al., 2003, 2004). It has also been described in *S. dysenterieae* serotype 1 and *S. flexneri* serotypes 3a and 3b (Baker et al., 2015; Njamkepo et al., 2016; Parajuli et al., 2019). On the other hand, we described the presence of the *dfrA14* gene in 79 out of 80 strains isolated in 2008–2009, differentiating from strains isolated before that period. This marker is coded in the pABC-3 plasmid in the *sul2/strA*::*dfrA14/strB* gene cluster (Miranda et al., 2016).

Based on the results of this study, we considered CHL resistance in MDR strains as a predictive phenotypic marker of the presence of SRL PAI, which pointed us to look at this marker more closely. The 2000–2010 report from Foodnet at CDC highlighted a high level of MDR *S. sonnei* strains in United States (Shiferaw et al., 2012). It is noteworthy that, from the 954 *S. sonnei* strains analyzed in that period, only 10 isolates (1%) included the CHL resistance phenotype, suggesting a low frequency of SRL-positive strains.

The *S. sonnei* strains circulating in Europe have diverse MDR patterns. Surprisingly, in the 18 years of surveillance in Belgium (Vrints et al., 2009), non-CHL resistance was detected in almost 5,000 isolates, suggesting the absence of SRL-positive strains. On the other hand, a study conducted in England and Wales characterized 341 *S. sonnei* isolated during 2015 by WGS (Sadouki et al., 2017). From that collection, only 8 strains (2.3%) appeared to carry both elements, the SRL PAI and the pABC-3 plasmid, based on the presence of sequence-predicted resistance genes. These strains could have been imported by travelers (Kotloff et al., 2018). Besides these, studies conducted by Baker et al. (2018) also demonstrated the presence of SRL and plasmid pABC-3 not only in *S. sonnei* but also in *S. flexneri* 2a strains.

In China, it is challenging to realize the presence of the SRL PAI in *S. sonnei* strains since CHL is not included in the AST. However, in a 10-year surveillance in Jiangsu (2002–2011), atypical class 1 integron was detected in 168 of 340 *S. sonnei* isolates (49.4%) harboring *bla*_OXA–1_-*aadA1* gene cassettes (Gu et al., 2017; Kang et al., 2019). As mentioned, this integron is part of the SRL locus, suggesting the presence of SRL PAI in strains isolated from this region. Indeed Zhang et al. (2014) described the presence of SRL in an epidemic clone of *S. flexneri* MDR strain, carrying genes for resistance against streptomycin, ampicillin, chloramphenicol, and tetracycline.

A completely different picture was observed with the Chilean *S. sonnei* strains. Since 1998, a high resistance to CHL has been reported (Fullá et al., 2005; Toro et al., 2005; Marcoleta et al., 2013). The present study detected 55.9% (195/349) of CHL-resistant strains among isolates from the 1995–2013 period, 191 of which possess the SRL PAI, thus correlating it as the most critical genetic element associated with CHL resistance.

The landscape of *S. sonnei* in Latin America was recently described by Baker et al. (2017). From WGS studies, it is possible to infer the circulation of SRL PAI in this region. Based on supplementary data analysis, we confirmed that 79 out of the 323 strains characterized by the PulseNet Latin American group at least harbored the SRL locus. The distribution of predicted SRL-positive strains was 46/48 Peruvian strains, 16/31 Colombian strains, and 17/27 Chilean *S. sonnei* isolates. All these strains belonged to sublineage IIIa. The sequence analysis of Peruvian *S. sonnei* strains showed that 40 out of the 48 strains harbor the SRL locus and the *dfrA14* gene probably as part of pABC-3 plasmid (Miranda et al., 2016), while 6 out of the 48 seem to carry only the SRL locus. In that dataset, the oldest Peruvian isolates were obtained in 1999, indicating the simultaneous presence of SRL PAI and the pABC-3 plasmid in those strains. In Colombia, 2 and 14 strains were predicted to harbor SRL PAI and SRL/pABC-3 genetic elements, respectively. For the Chilean strains analyzed in that work, the authors had access to strains isolated in the period 2010–2011 from different areas of the country, and the presence of SRL and SRL/pABC-3 was predicted in 3 and 14 of them, respectively (Baker et al., 2017). It is very likely that more than one of these strains coincide with those included here in period D, where we described 21% SRL-positive strains and 43.8% of SRL/pABC-3 strains. Our results show the close phylogenetic relationship in sublineage IIIa of all the Chilean strains harboring the SRL PAI to be nearly related with the Peruvian and Colombian isolates, suggesting the interchange of strains among these countries (Figure 5).

The results of the current study show that SRL-positive strains have been circulating in Chile from 1995 onward, and *dfrA14* was not present until 2006 when a SRL/pABC-3-positive strain was isolated for the first time. After that, all the strains included in period C (2008–2009 period) harbored SRL/pABC-3 genetic elements. It should be noted that this group is formed by 61 strains from different areas of Región Metropolitana, and 15 were isolated in Antofagasta, showing the dissemination to a much wider geographical area of the country. It is not known why these strains spread massively in our country in those years. Even the diminished prevalence of *Shigella* after 2009 and the co-circulation of different clones later have not been resolved (Figure 1A).

Interestingly, the *S. sonnei* strains circulating in Brazil appear to be different clones. A study of 72 Brazilian strains isolated over 31 years from 1983 to 2014 suggests two prevalent subtypes that differ little; however, neither of them displays the MDR associated with the presence of SRL PAI (Seribelli et al., 2016). Although a more recent study showed that most *S. sonnei* strains isolated in northeastern Brazil were associated with azithromycin resistance, it seems that the SRL element is circulating as 20% of them were resistant to CHL (Medeiros et al., 2018).

Surveillance for MDR *S. sonnei* infections acquired from domestic and international sources allowed the isolation of two *S. sonnei* strains in Pennsylvania from patients infected during a international travel to Peru (Abelman et al., 2019), showing the spread of SRL-positive strains to North America. The predicted antimicrobial gene resistance markers harbored by those strains strongly suggest the presence of the same Latin American clone, including SRL PAI (*bla*_OXA–1_, *aadA1*, *tetB*, and *cat1*) and the plasmid pABC-3 (*strA*, *strB*, *sul2*, and *dfrA14*). The key marker is again CHL resistance; only these strains had the appropriate phenotype and the genetic markers for the presence of SRL PAI.

The increase in *Shigella* spp. MDR strains has been a cause for concern worldwide over the past two decades. The spread of class 1 and class 2 integrons has also been documented in Latin America (Barrantes and Achí, 2016). The emergence and successful spread of a particular MDR strain of *S. sonnei* biotype g carrying a class 2 integron, resistant to STR, SXT, and TET, was reported in several countries, including Australia, Senegal, Taiwan, Japan, Korea, and Iran (McIver et al., 2002; Oh et al., 2003; Gassama-Sowa et al., 2006; Seol et al., 2006; Ranjbar et al., 2007; Izumiya et al., 2009; Chang et al., 2011). Among the Chilean *S. sonnei* strains, we could detect the presence of class 2 integron to be circulating steadily just since 2011.

The World Health Organization has recommended fluoroquinolones and cephalosporins as the preferred drugs for the treatment of *Shigella* infections (Williams and Berkley, 2016, 2018). However, resistance to these antibiotics has been described. During 1998–2009, the nalidixic acid resistance rates in *S. sonnei* isolated from Asia and Africa have increased to 44%, whereas in Europe and America they were less than 3%. Consequently, the spread of ciprofloxacin-resistant *S. sonnei* has been increasing globally, apparently from South Asia (Gu et al., 2012; Chung The et al., 2016, 2019, 2021). In Chile, ciprofloxacin resistance was not detected during the period 1995–2013, but the presence of 9.5% of NAL^R^ strains among these 349 isolates, concentrated in a short time between 2011 to 2013, is of great concern. Two of these Chilean NAL^R^ strains were sequenced, thus finding the D87Y mutation in *gyrA*. Moreover, the latest report of ISP showed the emergence of CIP^R^ in Chilean *S. sonnei* strains to be at 2–3% (ISP, Boletín Vigilancia de Laboratorio, *Shigella* 2014–2018).

Our results regarding the emergence of NAL^R^ strains agree with reports from Latin American (Sati et al., 2019). Such surveillance provides evidence of the increasing prevalence of NAL^R^ *S. sonnei* in this region since 2011. This information supports the presence of class 2 integron and the MDR phenotype to TET, STR, SXT, TMP, and NAL, suggesting that the entry of NAL^R^ strains of *S. sonnei* is more likely due to global world trade and human travel (Chung The et al., 2016, 2021).

In summary, this work shows an overview of the resistance phenotypes associated with genetic determinants, emphasizing the MDR molecular mechanisms that have circulated in Chile during the period 1995–2013. We were able to demonstrate the presence of a clone or variant of *S. sonnei* SRL-*dfrA14*, harboring the pABC-3 plasmid, which spread during the 2008–2009 period. After that, different clones harboring class 2 integron have emerged. Therefore, our results underscore the role of the SRL PAI and integrons as genetic elements associated with the MDR phenotype. On the other hand, the high similarity of the SRL sequences from representative strains of different periods would rule out that this element has affected by itself the spread of *S. sonnei* isolated during period C, 2008–2009. Even considering the limitations of the current study, the data presented in this work revealed the temporal dynamics of antimicrobial resistance in *S. sonnei* strains circulating in Chile, mainly determined by the widespread dissemination of MGE conferring MDR phenotypes. Since shigellosis is endemic in Chile, constant surveillance of antimicrobial resistance phenotypes and their genetic basis is a priority in order to contribute to the orientation of public health policies.

## Data Availability Statement

The datasets presented in this study can be found in online repositories. The names of the repository/repositories and accession number(s) can be found in the article/Supplementary Material.

## Author Contributions

CT and JS contributed to study design, data analysis, data interpretation, manuscript writing, and revision of the manuscript. DM contributed to data analysis, data interpretation, manuscript writing, figures design, and revision of the manuscript. JU contributed to data analysis, data interpretation, and revision of the manuscript. JD contributed to data analysis and revision of the manuscript. LC and RC contributed to data acquisition and data analysis. TH and CG contributed to data acquisition, data analysis, and data interpretation. GH and MU contributed to study design, data analysis, data interpretation, and revision of the manuscript. CT was the principal investigator at the FONDECYT grant that funded this work. All authors contributed to the article and approved the submitted version.

## Conflict of Interest

The authors declare that the research was conducted in the absence of any commercial or financial relationships that could be construed as a potential conflict of interest.

## Publisher’s Note

All claims expressed in this article are solely those of the authors and do not necessarily represent those of their affiliated organizations, or those of the publisher, the editors and the reviewers. Any product that may be evaluated in this article, or claim that may be made by its manufacturer, is not guaranteed or endorsed by the publisher.

## Footnotes

1
https://www.cdc.gov/pulsenet/pathogens/protocols.html

2
https://github.com/tseemann/shovill

3
https://link.springer.com/chapter/10.1007%2F978-3-642-37195-0_13

4
https://github.com/tseemann/snippy

5
https://cge.cbs.dtu.dk/services/ResFinder/

6
https://www.cdc.gov/narms/resources/glossary.html
