# Supplementary material for: Antimicrobial Resistance Dynamics in Chilean Shigella sonnei Strains Within Two Decades: Role of Shigella Resistance Locus Pathogenicity Island and Class 1 and Class 2 Integrons
Source: Front Microbiol. 2022 Feb 4;12:794470. doi: 10.3389/fmicb.2021.794470 (PMC8854971; doi:10.3389/fmicb.2021.794470)
Supplement: Supplementary file 1 [file Table_1.DOCX]

Supplementary Table 1. Primers used in this work

|  | **Primer names** | **Primer sequences (5’-3’)** | **Amplicon Size (bp)** | **Source** |
| --- | --- | --- | --- | --- |
| Class 1 integron integrase | Int1F Int1R | ACAGGCTTATGTCCACTGGG  CTGGTCCTTCAGCCACCAT | 508 | *This work* |
| Class 2 integron integrase | Int2F Int2R | tatccctgcaagcaagccta  acgccttgtaagttgtcgtc | 309 | *This work* |
| SRL I  *serX* neighbouring region | SerX-FS SerX-R | GAGTGGTTAATCACGCGTTG  CACCATTGGGTATCGAACGT | 1059 | *This work* |
| SRL PAI integrase | intSRL-F intSRL-R | GCGGGGGAAATGGGTTTTTC  TAACTGCCTCCAGGGCATTG | 541 | *This work* |
| SRL II  5’ end of SRL PAI | SerX-FS intSRL-R | GAGTGGTTAATCACGCGTTG TAACTGCCTCCAGGGCATTG | 1510 | *This work* |
| SRL PAI *orf58* | orf58-F orf58-R | GTCCGCCCTCAGTTGTTGTA  CTGAGCGGTATCATCAGCGT | 497 | *This work* |
| SRL III  3’ end of SRL PAI | orf58-F  SerX-R | GTCCGCCCTCAGTTGTTGTA  CACCATTGGGTATCGAACGT | 1950 | *This work* |
| *dfrA1* | dfrA1-F  dfrA1-R | GGAGTGCCAAAGGTGAACAGC  GAGGCGAAGTCTTGGGTAAAAAC | 367 | Toro et al*.,* 2005 |
| *dfrA8* | dfrA8-F  dfrA8-R | GAGCTTCCGGGTGTTCGTGAC  CTTCCATGCCATTCTGCTCGTAGT | 247 | Toro et al., 2005 |
| *dfrA14* | dfrA14-F  dfrA14-R | TTAACCCAGGATGAGAACCT  CGATTGCATAGCTTTGTTAA | 510 | Miranda et al, 2016 |
| *bla*_OXA-1_ | oxa-F  oxa-R | ACTGTCGCATCTCCATTATTTGA  ATCGCATTTTTCTTGGCTTTTAT | 713 | Toro et al*.,* 2005 |
| *aadA1* | aad1-F  aad1-R | TATCCAGCTAAGCGCGAACT  ATTTGCCGACTACCTTGGTG | 490 | *This work* |
| *cat* | cat-F  cat-R | AACGACCCTGCCCTGAAACC  TTGCGCCGAATAAATACCTG | 1003 | Toro et al*.,* 2005 |
| *tetB* | tet-3  tet-4 | CTGGATTACTTATTGCTGGCTTTTT  CACCTTGCTGATGACTCTTTGTTTG | 751 | Toro et al., 2005 |
| *qacEΔ1* | qacEΔ1-F  qacEΔ1-R | ATCGCAATAGTTGGCGAAGT  CAAGCTTTTGCCCATGAAGC | 226 | Di Conza et al. 2002 |
